# IglC and PdpA Are Important for Promoting *Francisella* Invasion and Intracellular Growth in Epithelial Cells

**DOI:** 10.1371/journal.pone.0104881

**Published:** 2014-08-12

**Authors:** H. T. Law, Aarati Sriram, Charlotte Fevang, Eli B. Nix, Francis E. Nano, Julian Andrew Guttman

**Affiliations:** 1 Department of Biological Sciences, Simon Fraser University, Burnaby, British Columbia, Canada; 2 Department of Biochemistry and Microbiology, University of Victoria, Victoria, British Columbia, Canada; University of Alabama, France

## Abstract

The highly infectious bacteria, *Francisella tularensis*, colonize a variety of organs and replicate within both phagocytic as well as non-phagocytic cells, to cause the disease tularemia. These microbes contain a conserved cluster of important virulence genes referred to as the *Francisella* Pathogenicity Island (FPI). Two of the most characterized FPI genes, *iglC* and *pdpA*, play a central role in bacterial survival and proliferation within phagocytes, but do not influence bacterial internalization. Yet, their involvement in non-phagocytic epithelial cell infections remains unexplored. To examine the functions of IglC and PdpA on bacterial invasion and replication during epithelial cell infections, we infected liver and lung epithelial cells with *F. novicida* and *F. tularensis* ‘Type B’ Live Vaccine Strain (LVS) deletion mutants (Δ*iglC* and Δ*pdpA*) as well as their respective gene complements. We found that deletion of either gene significantly reduced their ability to invade and replicate in epithelial cells. Gene complementation of *iglC* and *pdpA* partially rescued bacterial invasion and intracellular growth. Additionally, substantial LAMP1-association with both deletion mutants was observed up to 12 h suggesting that the absence of IglC and PdpA caused deficiencies in their ability to dissociate from LAMP1-positive *Francisella* Containing Vacuoles (FCVs). This work provides the first evidence that IglC and PdpA are important pathogenic factors for invasion and intracellular growth of *Francisella* in epithelial cells, and further highlights the discrete mechanisms involved in *Francisella* infections between phagocytic and non-phagocytic cells.

## Introduction

The intracellular bacteria *Francisella tularensis* cause the zoonotic disease in humans and other mammals, called tularemia. *F. tularensis* have multiple subspecies including ssp. ‘type A’ *tularensis*, ssp. ‘type B’ *holarctica* and ssp. *mediasiatica*. Though *F. novicida* has been considered a fourth subspecies because of its high genomic identity (>97%) with *F. tularensis* [1]–[4], this taxonomic classification remains controversial due to the distinct evolutionary and ecological profiles between the two bacteria [5]–[7]. Both *F. novicida* and *F. tularensis* can enter hosts through multiple routes including the intestinal and respiratory tracts as well as through exposure from infected animals or arthropod vectors. Once internalized, these bacteria colonize various organs, including the lungs and liver [8]–[16]. *F. tularensis* cause disease from exposure to as few as 10 bacteria, which can result in mortality rates as high as 60% if left untreated [17], [18] whereas *F. novicida* causes human-like tularemia in mice with as few as 50 bacteria [15], [19]. *F. tularensis* LVS can causes disease in mice and can infect human cells in culture [15], [16], [20]. All of these microbes infect phagocytic and non-phagocytic cells [15], [16], [20], [21], and contain similar sets of virulence factors [22]–[24]. Because *F. tularensis* has the potential for aerosolization and is classified by the USA Centers for Disease Control and Prevention as a ‘Category A’ select agent [25], requiring BCL-3 facilities for experimentation, much of the research on *Francisella* have used *F. novicida* and *F. tularensis* LVS (BCL-2) as surrogate pathogens to investigate the genetics, biochemistry and pathogenesis of *Francisella* [15], [26]–[31].

The *Francisella* pathogenicity island (FPI) is a highly conserved ∼31 kb region, comprising 16 to 19 protein-coding genes, within the bacterial chromosome. It is thought to exist as a single copy in *F. novicida*, but is duplicated in all *F. tularensis* species [32]. Evidence has shown that deletion of a single FPI gene in *F. tularensis* does not significantly diminish its virulence suggesting that both alleles are phenotypically alike [33]. Though most FPI genes are unique to the *Francisellaceae* family and remain poorly defined, a subset of genes, including *iglA*, *iglB*, *dotU*, and *vgrG,* share limited homology with core structural components of a Type VI secretion system (T6SS) [27], [34]–[36]. A thorough study by Bröms and colleagues examining *dotU*, *iglC*, *iglG*, and *vgrG* suggest that all four genes are needed for the delivery of virulence factors into host macrophages [37].

A commonly associated function of the FPI is its involvement in the replication of *Francisella* within host macrophages. During the initial intracellular stage of these infections, *Francisella* transiently reside within phagosomes and associate with early and late stage endosomal markers [38], [39]. In order to evade lysosome-mediated killing, *Francisella* can escape these vacuoles in as little as 15 min [40]; by 4 h, ∼90% of the bacteria are already present in the host cytosol [41], [42]. Several studies have demonstrated that most FPI-encoded proteins, including IglC and PdpA, are essential for bacterial escape from the phagosomal compartment and/or bacterial replication [27], [32], [43]–[47]. Although we have yet to fully understand how these proteins interact with host cells, recent evidence suggests that IglC, PdpA, and six other FPI-encoded proteins are translocated into the cytoplasm of phagocytes and their delivery is dependent on the expression of *Francisella* T6SS genes [37]. Despite the importance these T6SS genes play in intracellular replication, their expression does not appear to significantly influence the uptake of *Francisella* into macrophages [43], [48]–[50].

Non-phagocytic epithelial cell infections are considered to be important for the progression of disease as *Francisella* colonize and replicate within epithelial cells both *in vitro* and *in vivo* [16], [21], [51]–[53]. Although several organs are known to be colonized by these bacteria [8]–[14], two primary sites of *Francisella* colonization are the lungs and liver. The lungs are susceptible to infection through aerosol delivery, requiring the low infectious doses to trigger disease [54]. Once inside the lungs, *F. tularensis* can infect alveolar type II epithelial cells, which are considered well positioned for the pathogen to enter the circulation as they reside near the endothelium [16], [55]. The livers of *Francisella* infected animals have been known for decades to be sites of bacterial colonization [56], [57]. Within the livers of infected mice, ∼12% of the cells are colonized by these microbes; >90% of which are hepatocytes [15]. We have previously shown that *Francisella* predominantly invade hepatocytes by usurping the host clathrin-mediated endocytic (CME) machinery [15]. Although the precise bacterial mechanisms that *Francisella* use to hijack the host endocytic machinery has remained elusive, viruses [58]–[60] and other invasive pathogens including *Listeria monocytogenes* and *Yersinia pseudotuberculosis* rely on virulence factors encoded within clusters of pathogenic genes to trigger bacterial internalization *via*. CME [61], [62].

Despite recent work that has alluded to the importance of non-macrophage infections in the *Francisella* disease process [63], [64], the study of *Francisella* remains primarily focused on phagocytic cell infections. Given that a previous report has indicated that the FPI likely plays a predominant role in the pathogenesis of *Francisella* in epithelial cells [65], we examined the involvement of two FPI genes in *Francisella* invasion and replication during liver and lung epithelial cell infections. We report that *iglC* and *pdpA* are required for efficient bacterial invasion and intracellular proliferation during non-phagocytic epithelial cell infections.

## Experimental Procedures

### Bacterial and growth conditions

*F. novicida* strain Utah 112, deletion mutants Δ*iglC*, Δ*pdpA*, and their gene complements (Δ*iglC*::*iglC-*FLAG Km^R^ [Δ*iglC*::*iglC*], Δ*pdpA*::*pdpA*-FLAG Km^R^ [Δ*pdpA*::*pdpA*]) as well as *F. tularensis* ssp. *holarctica* Live vaccine strain [LVS], Δ*pdpA*, and Δ*pdpA*::*pdpA* Hyg^R^ were supplied by Dr. Francis Nano from the University of Victoria. The detailed description of the *F. tularensis* LVS Δ*pdpA* mutant will be described elsewhere (Nix et al, in preparation). In brief, the two *pdpA* alleles in *F. tularensis* LVS were sequentially removed using the approach previously used to remove the one *pdpA* allele from *F. novicida* [26]. Genetic complementation was accomplished by introducing a copy of *pdpA* on a derivative of plasmid pMP831 [66]. *F. tularensis* LVS Δ*iglC* and its complement *F. tularensis* LVS Δ*iglC*::*iglC* Km^R^ were kindly provided by Dr. Anders Sjostedt. All bacteria were grown according to the procedures described previously [36], [47], [48], [67].

### Bacterial titre

To estimate the amount of bacterial growth at 24 h post-infection (PI), 10-fold serial dilutions of the *Francisella* cultures were made in Tryptic soy broth (TSB) supplemented with 0.1% _L_-cysteine (TSBC). These were performed by plating 10 µL of diluted cultures that ranged from 10^−1^ to 10^−10^ onto Tryptic soy agar (TSA) supplemented with 0.1% _L_-cysteine (TSAC) plates (for *F. novicida*) or Chocolate blood agar [TSAC and 5% defibrinated horse blood] (CBA) plates (for *F. tularensis* LVS). When appropriate, bacteria were grown in media containing 10 or 15 µg mL^−1^ of kanamycin or 100 µg mL^−1^ of hygromycin B. Plates were incubated overnight at 37°C (for *F. novicida*) or for up to 3 days (for *F. tularensis* LVS), at 37°C prior to counting colony forming units (CFUs).

### Cell cultures and infections

Murine hepatocytes, BNL CL.2 cells (ATCC; TIB-73) and human lung epithelial A549 cells (ATCC; CCL-185) were respectively cultured in high-glucose Dulbecco’s Modified Eagle’s Medium (DMEM) [Thermo] and Kaghn’s F-12 media [Thermo] both containing with 10% fetal bovine serum (FBS) and incubated at 37°C with 5% CO_2_. These cells were seeded into 24-well plates or onto #1.5 glass coverslips, grown to confluence, then infected with *F. novicida* at a multiplicity of infection (MOI) of 100∶1 for up to 48 h as done previously [15]. For *F. tularensis* LVS studies, the cells were infected at an MOI of 200∶1 and immediately centrifuged for 5 min (300×g) at room temperature (RT), as previous described [68]. In this manuscript this is referred to as the 0 h time-point. Antibiotic selection was maintained over the course of the infection by supplementing the cell culture media with appropriate antibiotics. All infections proceeded in a humidified chamber at 37°C with 5% CO_2_ and were only placed at RT during inoculation, washes, and at the experimental endpoint. At the appropriate time-points, samples were washed 6 times with PBS +/+ (Dulbecco’s PBS containing 0.0133% calcium and 0.01% magnesium) [Sigma] both before and after extracellular bacterial-killing using fresh infection media supplemented with 30 (for LVS) or 100 (for *F. novicida*) µg mL^−1^ of gentamicin [15], [55]. When indicated, a lower concentration of gentamicin (10 µg mL^−1^) was subsequently applied to prevent the growth of extracellular bacteria. Before commencing gentamicin protection assays or immunofluorescence staining, all wells were washed 3 times with PBS +/+.

### Gentamicin protection assays

Gentamicin protection assays (4 h invasion assays) were performed following the procedures previously used by our laboratory [15]. To summarize, the samples were washed 6 times with PBS +/+ then incubated with gentamicin at 3 h post inoculation (PI) to kill any extracellular bacteria. At all of the experimental endpoints, uninfected and *F. novicida* infected cells were lysed with 1% Triton-X in PBS. For *F. tularensis* LVS infections, cells were lysed with sterile ddH_2_O because the lysis reagent prohibited the formation of visible colonies. The lysates were serially diluted with TSBC and then plated onto either TSAC (for *F. novicida*) or CBA (for *F. tularensis* LVS). Solid agar plates containing appropriate antibiotics were used when necessary and were subsequently incubated at 37°C.

To compare the intracellular growth profiles of wild-type and mutant *Francisella* between 4 h and 24 h PI, we allowed *Francisella* to invade epithelial cells for 3 h, washed the samples multiple times and treated them with gentamicin for 1 h. The samples were then either lysed immediately, to measure the initial amount of intracellular bacteria, or replaced with fresh media containing gentamicin (10 µg mL^−1^), thus permitting the invaded bacteria to replicate for up to 24 h PI. Bacterial replication was measured during the late stages (24–48 h PI) of infection by allowing *Francisella* to infect epithelial cells for 22 h, washing the samples with PBS, then adding gentamicin to kill uninvaded bacteria for 2 h. At 24 h PI, samples were either lysed to quantify intracellular bacteria or allowed to replicate further within epithelial cells for an additional 24 h in the presence of low concentrations of gentamicin.

### Differential bacterial immunolabeling

The method used for differential (inside/outside) bacterial labeling was based on previously published work [31]. To immunolocalize extracellular bacteria, 3% paraformaldehyde-fixed/non-permeabilized samples were washed with PBS, blocked with 5% normal goat serum (NGS) at RT for 20 min, and stained using a 1∶1000 dilution of rabbit anti-*Francisella* antibody [69] in PBS containing 0.1% BSA (PBS/BSA) supplemented with 1% NGS for 1 h at 37°C. Subsequently, the samples were washed with PBS/BSA and treated with 1 µg mL^−1^ of Alexa Fluor 594 goat anti-rabbit IgG (H+L) antibody (in PBS/BSA+1% NGS) for 1 h at 37°C. After washing with PBS/BSA, the samples were permeabilized with 0.2% Triton X-100 in PBS for 15 min, washed with PBS/BSA, and blocked with 5% NGS for an additional 20 min at RT. The samples were then treated with the primary rabbit anti-*Francisella* antibody [diluted 1∶1000 in PBS/BSA containing 0.05% Tween-20 (TPBS/0.1% BSA) and supplemented with 1% NGS] for 1 h, washed with TPBS/BSA, incubated with 1 µg mL^−1^ Alexa Fluor 488 conjugated goat anti-rabbit IgG (H+L) antibodies prepared in TPBS/BSA+1% NGS for 1 h at 37°C, washed with TPBS/BSA, and mounted using ProLong Gold antifade reagent with DAPI. This procedure labels intracellular bacteria green and extracellular bacteria (red and green in merged panels). Unless otherwise stated, all washes were repeated 3 times and lasted a total of 30 min.

To quantify the proportion of intracellular bacteria and lysosomal-associated membrane protein 1 (LAMP1), all samples were infected for 3 h then treated with gentamicin for 1 h. For 8, 12, and 24 h time-points, the infections were allowed to proceed in the presence of gentamicin (10 µg mL^−1^) until the experimental endpoints. Localization of *Francisella* required the use of mouse anti-*Francisella* 2H1 antibodies (1∶100) [Immunoprecise Antibodies] in combination with Alexa Fluor 350 and 594 goat anti-mouse IgG (H+L) antibodies to discriminate between intracellular (red and blue overlap) and extracellular (blue) bacteria. Following permeabilization of cell membranes, rat anti-LAMP1 1D4B antibodies (1∶50) [Developmental studies hybridoma bank] were used in combination with either Alexa Fluor 488 goat anti-rat (1∶100) to localize LAMP1. These coverslips were mounted with the ProLong Gold antifade reagent.

### Image and statistical analysis

A Leica DM4000B inverted fluorescence microscope attached to an Angstrom Grid Confocal system [Quorum Technologies] was used to view the samples and Metamorph Software was used to capture the images. ImageJ version 1.44i (http://imagej.nih.gov/ij) was used to count the number of invaded bacteria from images captured by either epifluorescence or structured illumination microscopy. Adobe Photoshop CS5 was used to process immunofluorescence images without changing the integrity of the data. To calculate the population of cells in each image, we semi-automatically measured the number of DAPI-stained cell nuclei using the “nucleus counter” plug-in, which is part of the McMaster Biophotonics Facility ImageJ bundle (http://www.macbiophotonics.ca/downloads.htm). In the nucleus counter plug-in, the following parameters were adjusted to tally the number of nuclei in each image [taken at 40X magnification]: 2000, ‘Smallest Particle Size’; 6000, ‘Largest Particle Size’, Mean 3×3, ‘Smooth method’; enabled, ‘Watershed filter’. In case there were any miscounted nuclei or multi-nucleated cells, each image was further reviewed by overlaying the DAPI-stained image with the phase-contrast image. Within the population of adherent hepatocytes, infected and uninfected cells were manually tallied. Each infected cell was given a score of ‘1’ based on the presence of *F. novicida* within the cell boundaries, whereas each uninfected cell that had an absence of intracellular *F. novicida* was given a score of ‘0’. The proportion of infected cells was calculated based on the number of infected cells divided by the total number of cells in each image. To determine whether *Francisella* was within a LAMP1 containing vacuole, we looked for bacteria that were at least 50% surrounded or completely co-localized with LAMP1.

**Figure 1:**
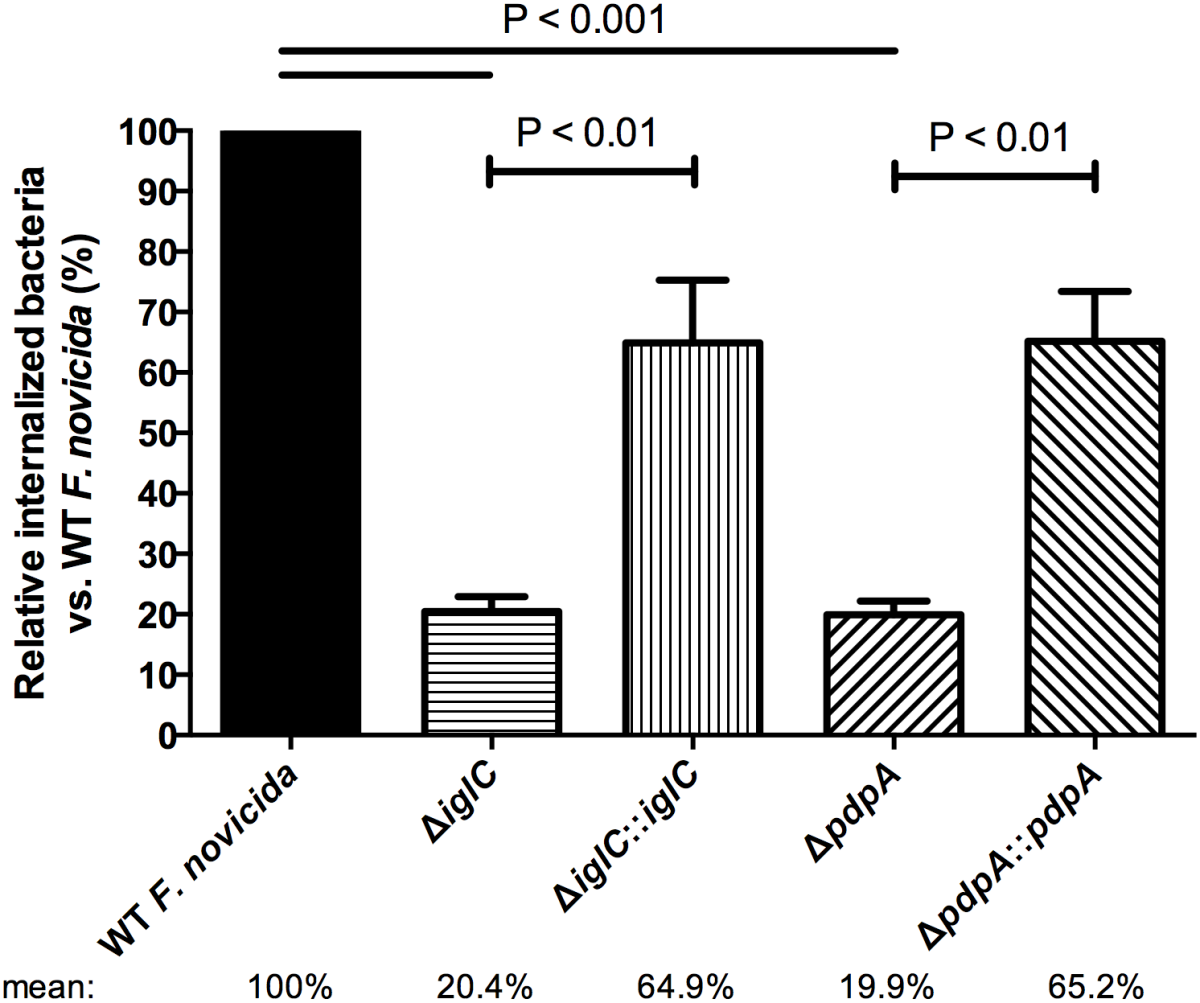
Deletion of genes encoding IglC and PdpA perturb *F. novicida* invasion. Murine BNL CL.2 hepatocytes were infected with wild-type *F. novicida*, deletion mutants (Δ*iglC* and Δ*pdpA*) as well as complement strains (Δ*iglC*::*iglC* and Δ*pdpA*::*pdpA*) for 3 h. Subsequently, samples were washed and treated with gentamicin for 1 h. At 4 h PI, lysates were plated onto agar-containing media and bacterial colonies were enumerated the following day. Error bars, S.E.M. (n = 4).

**Figure 2:**
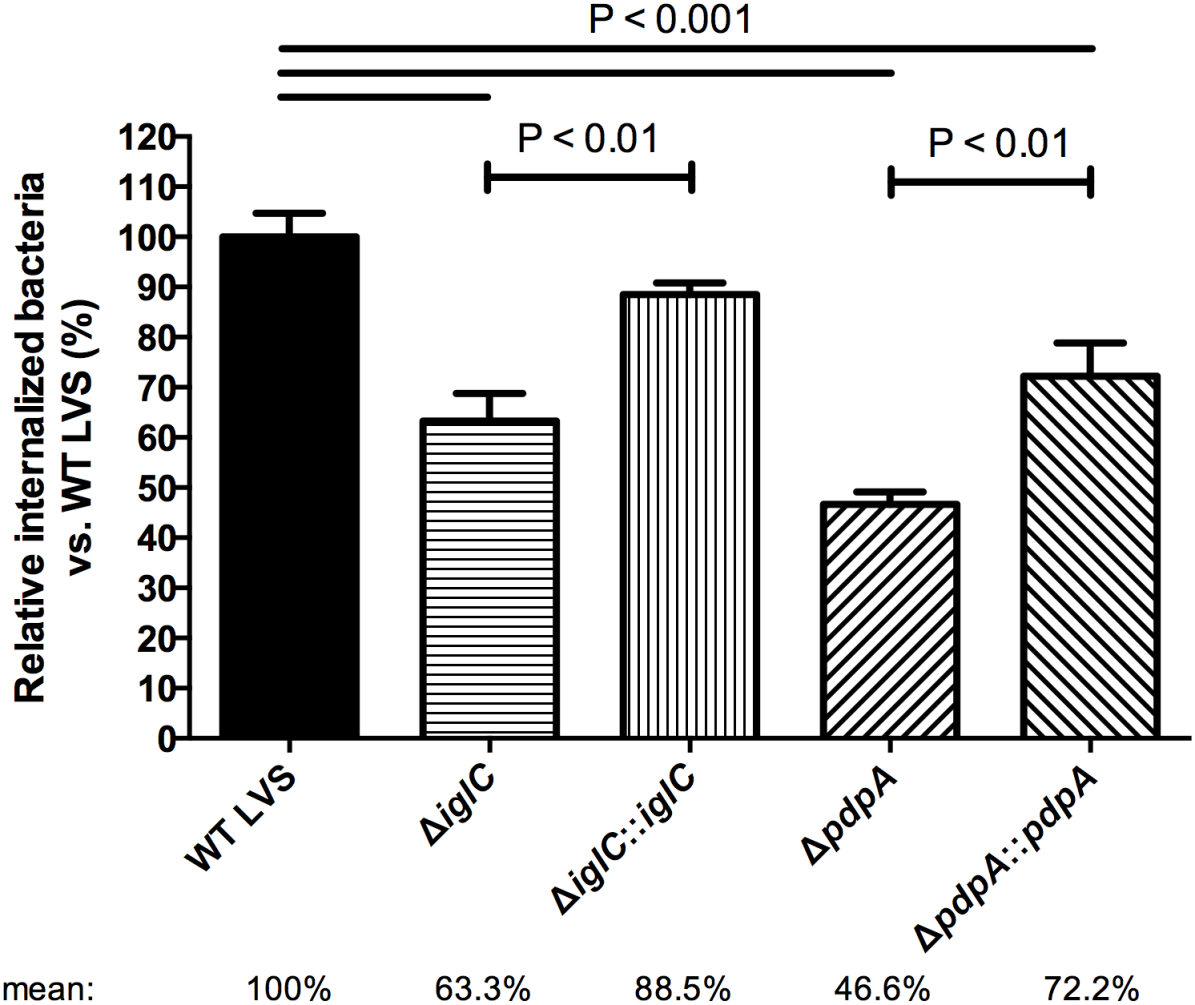
*F. tularensis* LVS Δ*iglC* and Δ*pdpA* mutants lung epithelial cell infections. Bacteria were centrifuged onto human A549 cells and allowed to invade for 3 h. To determine the amount of invaded bacteria, gentamicin protection assay (invasion assay) was performed at 4 h PI and bacterial titre was measured after a 3-day incubation. Error bars, S.E.M. (n = 3).

**Figure 3:**
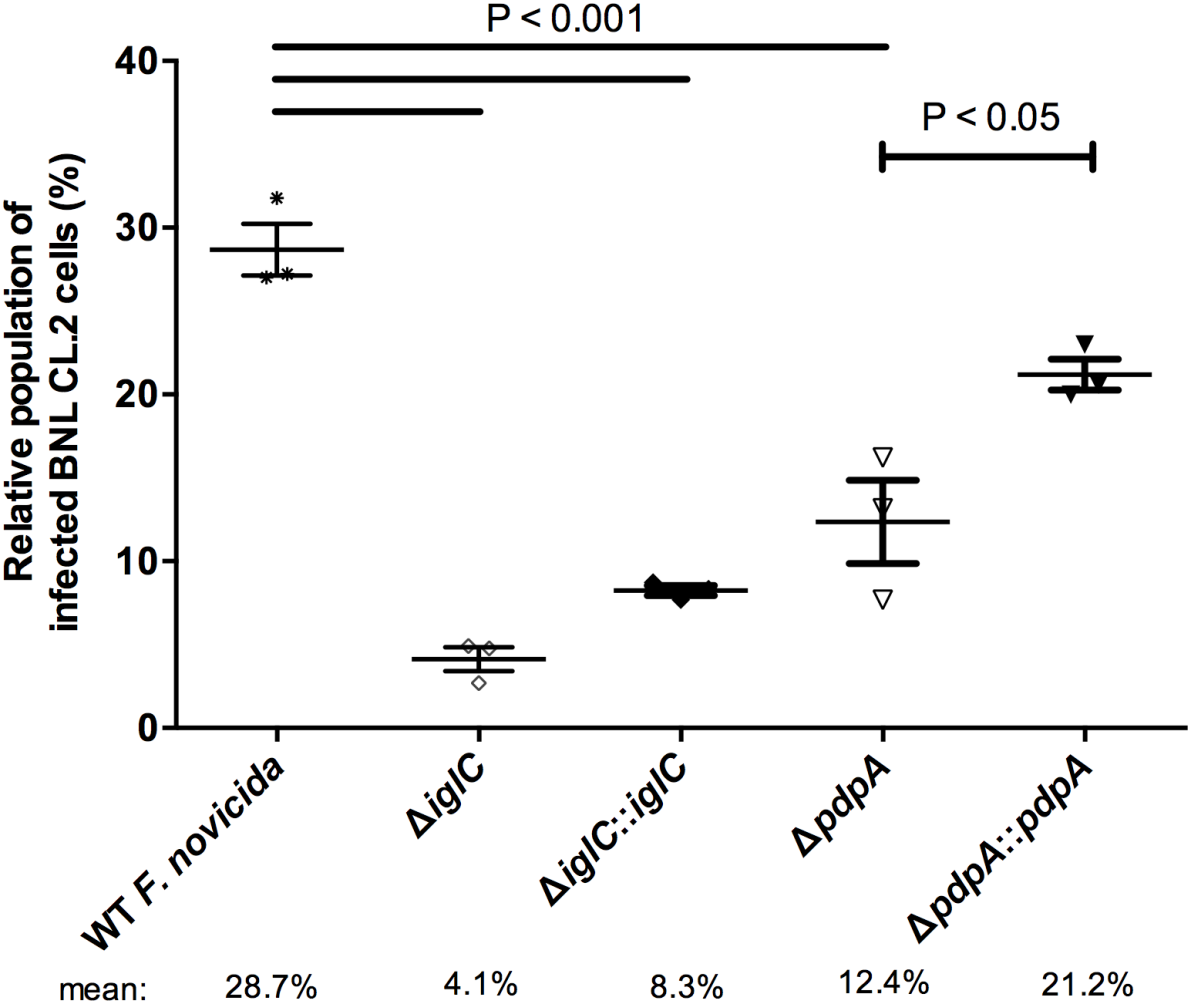
*F. novicida* lacking either IglC or PdpA reduces liver epithelial cell colonization. Samples were fixed at 24 h PI, differentially stained for intracellular and extracellular bacteria, and then visualized by fluorescence microscopy. The proportion of infected cells was tallied from over 1,000 cells. Cells containing one or more intracellular bacteria are considered ‘infected’. Error bars, S.E.M. (n = 3).

**Figure 4:**
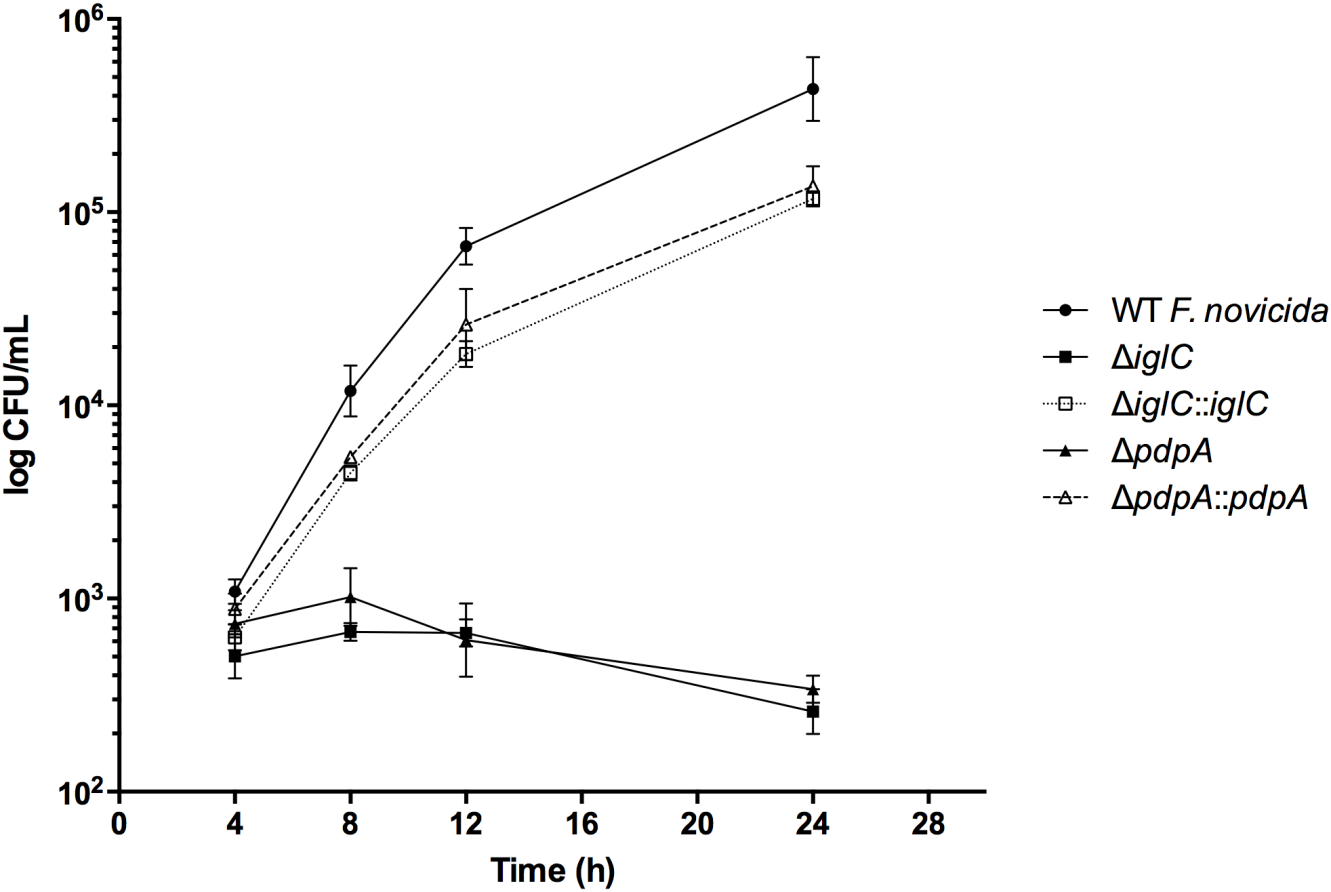
Intracellular growth kinetics of *F. novicida* mutants during hepatocyte infections. BNL CL.2 cells were infected with wild-type *F. novicida*, deletion mutants (Δ*iglC* and Δ*pdpA*) and their respective complements. Bacteria were allowed to invade for 3 h after which extracellular bacteria were rapidly washed with PBS and killed with 100 µg mL^−1^ of gentamicin for 1 h. Low concentrations of gentamicin (10 µg mL^−1^) remained in the media (to inhibit extracellular bacteria) until experimental endpoint. Intracellular bacteria were then released by lysing host cells, diluted with TSBC, and plated for bacterial enumeration. Error bars, S.E.M. (n = 3).

Statistical analysis was performed using Graphpad Prism 6 software. Statistical significance was calculated using one-way ANOVA followed by Bonferroni multiple comparison test for data in Fig. 1, 2 and 3. Data in Fig. 4 and 7 were analyzed by Two-way ANOVA followed by Bonferroni multiple comparison test, whereas for Fisher’s LSD test was used for data in Fig. 8.

## Results

### IglC and PdpA are required for efficient epithelial cell invasion

The involvement of the FPI genes *iglC* and *pdpA* has been examined during macrophage infections and was shown to be important for intracellular replication, but not for the uptake of *Francisella* into those cells [34], [48], [49]. To begin to investigate whether *iglC* and *pdpA* play a role in bacterial entry into epithelial cells, we evaluated the internalization of wild-type *F. novicida*, deletion mutants (Δ*iglC,* Δ*pdpA*), and their respective gene complemented strains during murine liver BNL CL.2 cell infections using gentamicin protection/invasion assays. These assays are common microbiological experiments used to quantify the amount of intracellular bacteria in an entire sample. Because minimizing the amount of intracellular bacterial replication is a general concern when performing these assays [21], [42], [70], we allowed *F. novicida* to contact and invade BNL CL.2 cells for 3 h before treating the samples with gentamicin for 1 h to kill the extracellular microbes. This time-point was selected because there are extremely few bacteria that are detectable by plating during infections lasting <3 h (unpublished data). Thus, at 4 h PI, host cells were lysed and intracellular bacteria were plated onto solid media. Using this procedure, we found a significant reduction of intracellular bacteria in samples infected with Δ*iglC* [20.4%] and Δ*pdpA* [19.9%] when normalized against wild-type *F. novicida* invasion [100%] (Figure 1). Invasion was significantly restored for both Δ*iglC*::*iglC* [64.9%] and Δ*pdpA*::*pdpA* [65.2%] when genes were re-introduced back into the microbe (Figure 1). Our bacterial deletion data at this early time-point suggest that IglC and PdpA influence *F. novicida* invasion into epithelial cells.

To determine whether these findings were *F. novicida* specific or if they were more broadly applicable to *Francisella* in general we ran similar assays using *F. tularensis* LVS infected A549 epithelial cells. We chose human lung A549 cells over BNL CL.2 cells for this assay primarily because of the low infections rates BNL CL.2 cells show at 4 h when infected with *F. tularensis* LVS. We found that *F. tularensis* LVS Δ*iglC* and Δ*pdpA* infected at a rate of 63.3% and 46.6% as compared to wild-type [100%] (Figure 2). Although this was significantly lower than wild-type *F. tularensis* LVS, it was not as dramatic as the decrease seen with *F. novicida* (Figure 1). A significant improvement in the numbers of intracellular bacteria was found when complemented strains were used (Figure 2), thus supporting our hypothesis that IglC and PdpA both play a role in the epithelial internalization process.

Given that our evidence showed that gene deletion of *iglC* and *pdpA* decreased the levels of intracellular bacteria, we then further studied this phenotype with *F. novicida* using fluorescence microscopy. These infections were performed for 24 h, instead of 4 h, because short infections resulted in <1% of total colonized hepatic cells when examined by microscopy. To distinguish infected and uninfected cells, hepatocytes were infected separately with wild-type *F. novicida,* deletion mutants (Δ*iglC* and Δ*pdpA*) and their respective complement strains. Samples were then fixed and labeled using differential bacterial staining, which allows extracellular (Figure S1; arrow) and intracellular bacteria (Figure S1; arrowhead) to be distinguished. After enumerating >1,000 cells, we found that wild-type *F. novicida* infected approximately 28.7% of BNL CL.2 cells (Figure 3), which is in-line with previous reports [15], [31]. The microscopic images point to both invasion and bacterial replication deficiencies during these mutant *F. novicida* infections, as extremely few bacteria were found in the cells in general. In cells that had intracellular bacteria, clusters of *F. novicida* that would be expected if bacterial replication were functional were not observed. Additionally, the population of cells colonized by Δ*iglC* [4.1%] and Δ*pdpA* [12.4%] mutants were significantly lower as compared to wild-type (Figure 3). Gene complementation of *pdpA* back into its respective deletion mutant significantly increased colonization [21.2%]. Although we observed a similar doubling in bacterial colonization for Δ*iglC*::*iglC* [8.3%] over the mutant strain, this increase was not significant (Figure 3). Together, our 24 h microscopic data provided similar results to the *F. novicida* gentamicin protection/invasion assays taken at 4 h, suggesting that *iglC* and *pdpA* genes are involved in both bacterial invasion and early stage replication within epithelial cells.

To further study the influence that *iglC* and *pdpA* have on intracellular replication, we infected mouse BNL CL.2 cells with wild-type *F. novicida* and mutants (Δ*iglC*, Δ*iglC*::*iglC,* Δ*pdpA*, and Δ*pdpA*::*pdpA*) and allowed them to invade for 3 h. The hepatocytes were then kept in gentamicin-containing media for up to 24 h, when the total intracellular bacterial loads were measured. We found that *F. novicida* deletion mutants Δ*iglC* and Δ*pdpA* did not show significant growth over the course of the infection (Figure 4), but rather, the population of intracellular Δ*iglC* and Δ*pdpA* declined marginally as early as 8 h PI (Figure 4). In contrast, wild-type *F. novicida* grew very rapidly from 4 to 8 h (Figure 4), with an average doubling time of 1.16 h, which slightly outpaced that of Δ*iglC*::*iglC* [1.53 h] and Δ*pdpA*::*pdpA* [1.41 h] (Table S1). By 12 h, the live growth was about 3-fold less and the doubling time for wild-type *F. novicida*, Δ*iglC*::*iglC*, and Δ*pdpA*::*pdpA* respectively increased to 4.44, 4.49, and 5.05 h (Table S1). These results demonstrate that the early stage replicative deficiencies seen with *F. novicida* Δ*iglC* and Δ*pdpA* at 4 h PI were maintained up to 24 h following epithelial cell infections.

**Figure 5:**
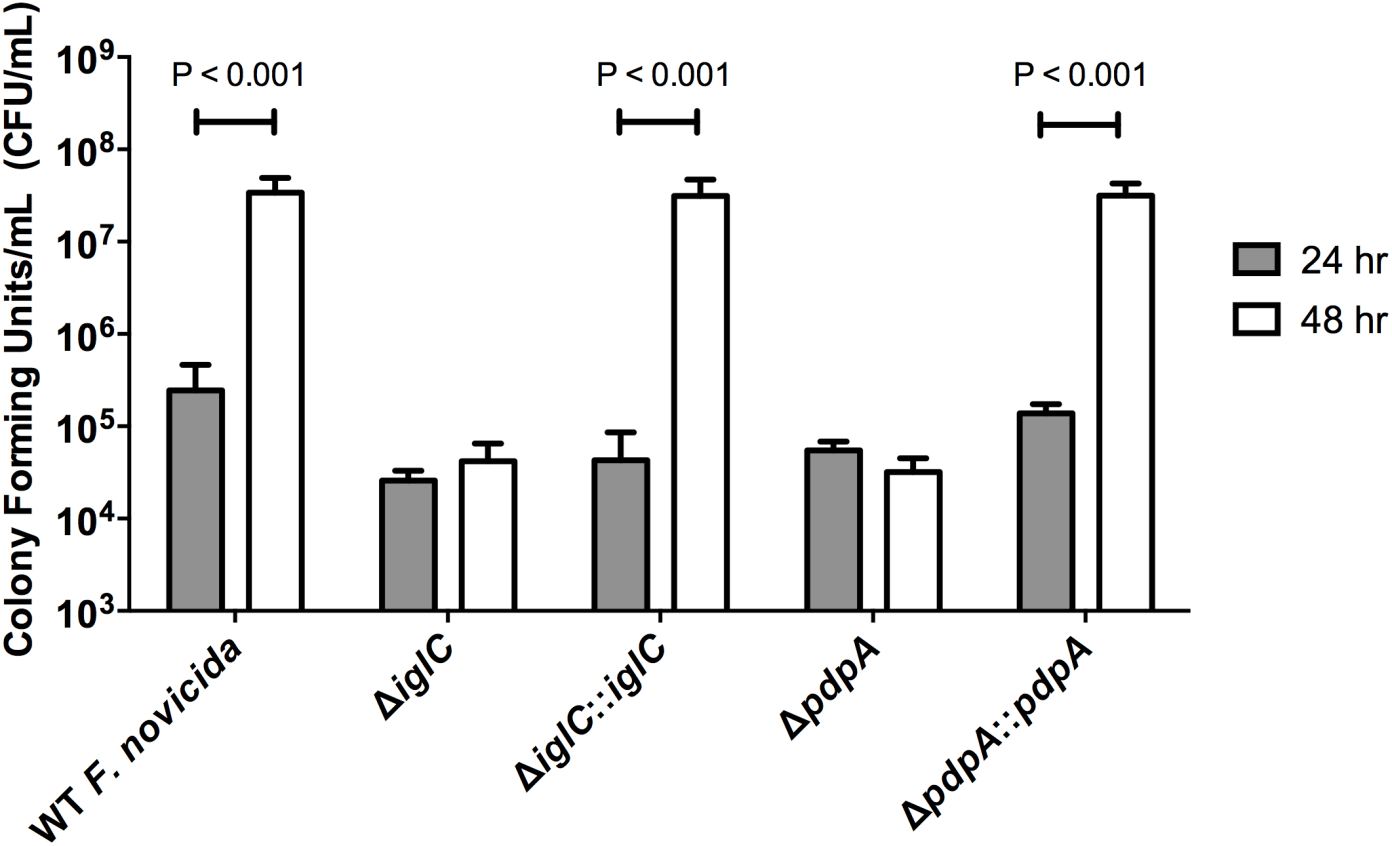
Intracellular bacterial replication is severely compromised when genes encoding *iglC* and *pdpA* are deleted. 24 h and 48 h gentamicin protection assays were performed on liver BNL CL.2 cells infected with wild-type *F. novicida*, deletion mutants (Δ*iglC* and Δ*pdpA*), and their respective complement strains (Δ*iglC*::*iglC* and Δ*pdpA*::*pdpA*). Samples were then treated with gentamicin starting from 22 h post-inoculation until the experimental endpoint. After host cells were lysed, the released bacteria were diluted and plated for CFU enumeration. Error bars, S.D. (n = 4).

**Figure 6:**
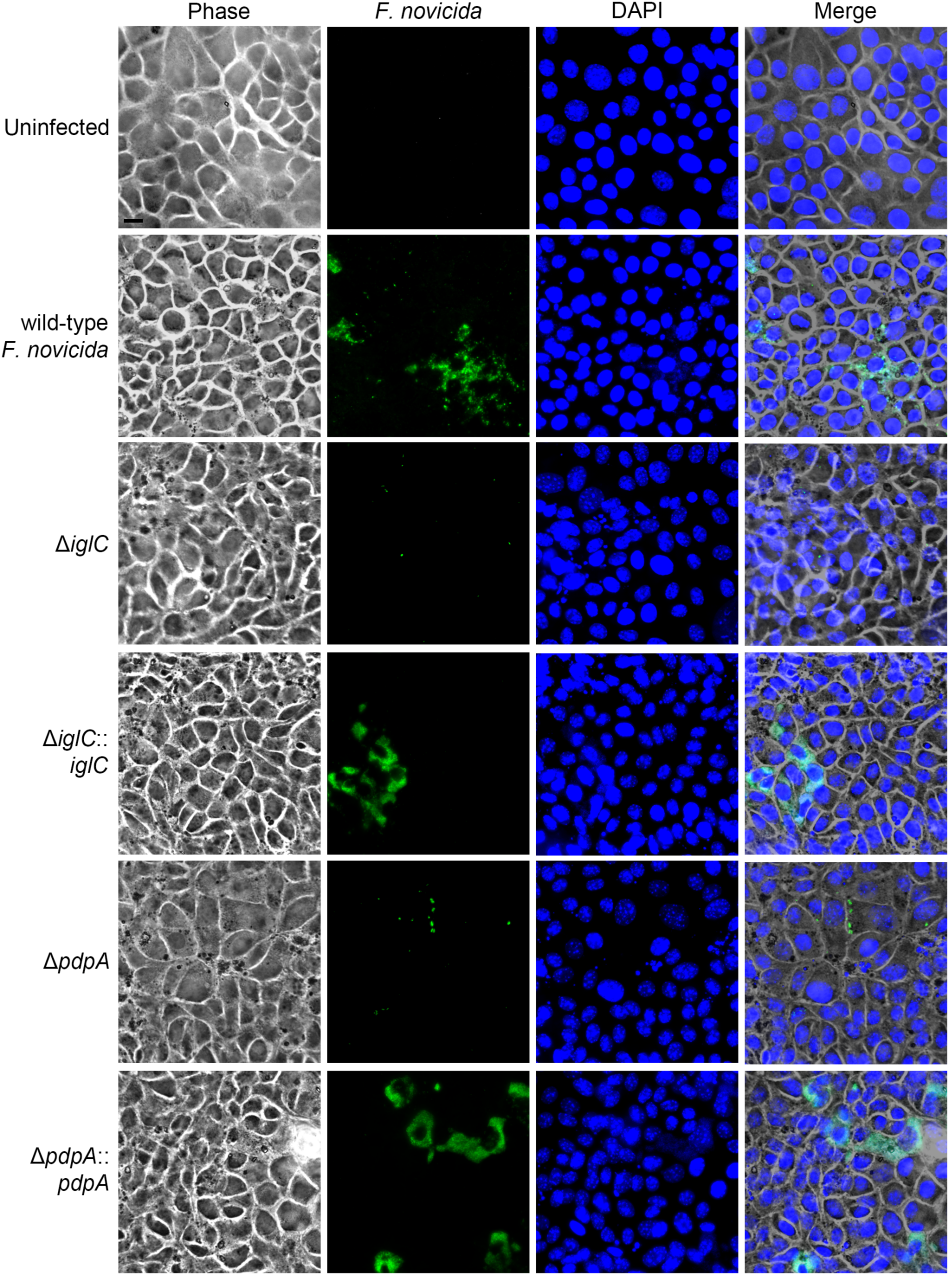
IglC and PdpA are essential for robust *F. novicida* growth within hepatocytes. Phase and fluorescence microscopic images were taken of BNL CL.2 cells infected with wild-type *F. novicida*, deletion mutants (Δ*iglC* and Δ*pdpA*), and complement strains (Δ*iglC*::*iglC* and Δ*pdpA*::*pdpA*) for 48 h. At 22 h post-inoculation, the samples were washed with PBS and replaced with media containing gentamicin to prohibit further bacterial invasion. *F. novicida* (green) and DNA (blue, DAPI) were stained in the fixed samples. Each image represents a ‘maximum intensity’ Z-projection comprising a stack through the cell body. Images taken by fluorescence and phase microscopy were merged together to illustrate the cell borders. Scale bar = 10 µm.

We also examined whether IglC and PdpA were important for bacterial replication during the late phase of its intracellular lifecycle. To test this, we compared the bacterial burden of *F. novicida* in BNL CL.2 cells at 24 h post-inoculation with a ‘prolonged’ 48 h infection in the absence of additional bacterial internalization by treating the cells with fresh media containing 100 µg mL^−1^ of gentamicin at the 22 h time-point, which was followed and maintained by 10 µg mL^−1^ of gentamicin from 24–48 h. We found that the amount of internalized wild-type *F. novicida* had increased by ∼100-fold in the final 24 h of infection (by the 48 h time-point) (Figure 5). During the same period, the Δ*iglC* and Δ*pdpA F. novicida* mutants both did not have a replicative burst (Figure 5). In contrast, gene complementation of Δ*iglC*::*iglC* and Δ*pdpA*::*pdpA* replicated to nearly wild-type *F. novicida* levels (Figure 5). Concurrently, we qualitatively assessed intracellular bacterial replication by fluorescence microscopy. Clusters of bacteria were found in colonized BNL CL.2 cells infected with wild-type *F. novicida,* as well as Δ*iglC*::*iglC* and Δ*pdpA*::*pdpA* complement strains; many of which were completely filled with bacteria (Figure 6). In contrast, *F. novicida*-filled cells were rarely (if ever) observed when hepatocytes were infected with Δ*iglC* and Δ*pdpA* deletion mutants and most infected cells contained only a small number of bacteria (Figure 6).

**Figure 7:**
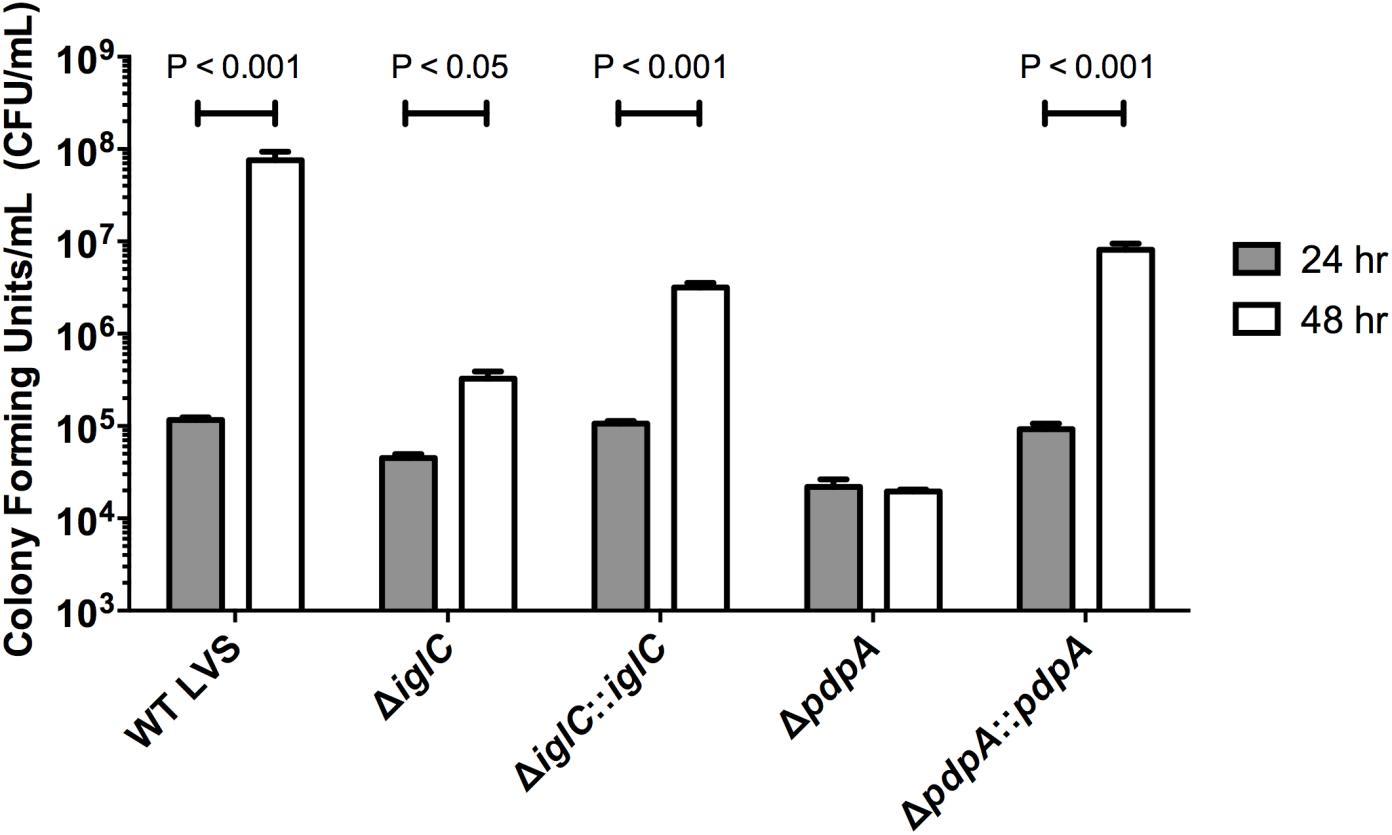
During the late intracellular phase, IglC and PdpA are necessary for efficient proliferation in lung epithelial cells. Human A549 cells were infected by wild-type *F. tularensis* LVS, Δ*iglC*, Δ*pdpA*, Δ*iglC*::*iglC* and Δ*pdpA*::*pdpA*. Intracellular bacteria were enumerated at 24 and 48 h time-points using gentamicin protection assay. At 24 h PI, the sample was switched to a low gentamicin concentration (10 µg mL^−1^) in order to inhibit growth of extracellular microbes. Intracellular bacteria were titred after they were released from host cells and serial diluted onto agar-containing media. Error bars, S.E.M (n = 3).

To determine whether this late-stage replicative phenotype was *F. novicida* specific we performed identical assays on *F. tularensis* LVS-infected A549 cells and found similar results; that *F. tularensis* LVS Δ*iglC* and Δ*pdpA* were both attenuated in their abilities to rapidly grow within infected cells at 48 h as compared to wild-type *Francisella* LVS (Figure 7). Interestingly, we noticed a gain in the amount of intracellular Δ*iglC* (Figure 7), which was not apparent when assayed with *F. novicida* (Figure 5). Increased bacterial levels were again seen with the gene complement strains Δ*iglC*::*iglC* and Δ*pdpA*::*pdpA* (Figure 7). Taken together this supporting evidence indicates that *iglC* and *pdpA* genes are important for efficient bacterial proliferation during all stages of epithelial cell infection.

### IglC and PdpA are crucial virulence factors for intracellular proliferation and LAMP1-positive FCV dissolution

Following bacterial entry in macrophages, *Francisella* are found enclosed within a membrane-bound compartment referred to as the *Francisella*-containing vacuole (FCV) [42]. The bacteria can reside within FCVs for up to few hours [70] until they escape into the cytoplasm where it is favorable for replication [42], [49]. Previous phagocytic cell studies have demonstrated that proficient escape from FCVs requires the presence of IglC [45], [46]. Yet, it is not known whether the same is true for PdpA. LAMP1 is a commonly used marker of FCVs and its presence or absence is indicative of the maintenance or dissolution of the FCV. Given that *iglC* and *pdpA* influence intracellular bacterial growth in both macrophages [32], [71] and epithelial cells, and because it is known that replication occurs after *Francisella* escape from the FCVs during macrophage infections [70], we investigated the temporal dynamics of LAMP1 around intracellular *F. novicida* at 4, 8, 12 and 24 h PI in order to determine whether *iglC* and *pdpA* affected its localization. To investigate this, we infected BNL CL.2 cells for 3 h with wild-type *F. novicida* as well as *iglC* and *pdpA* mutants and their complemented strains. Extracellular bacteria were then washed away and exposed to gentamicin for up to 24 h then processed for bacterial and LAMP1 localization.

**Figure 8:**
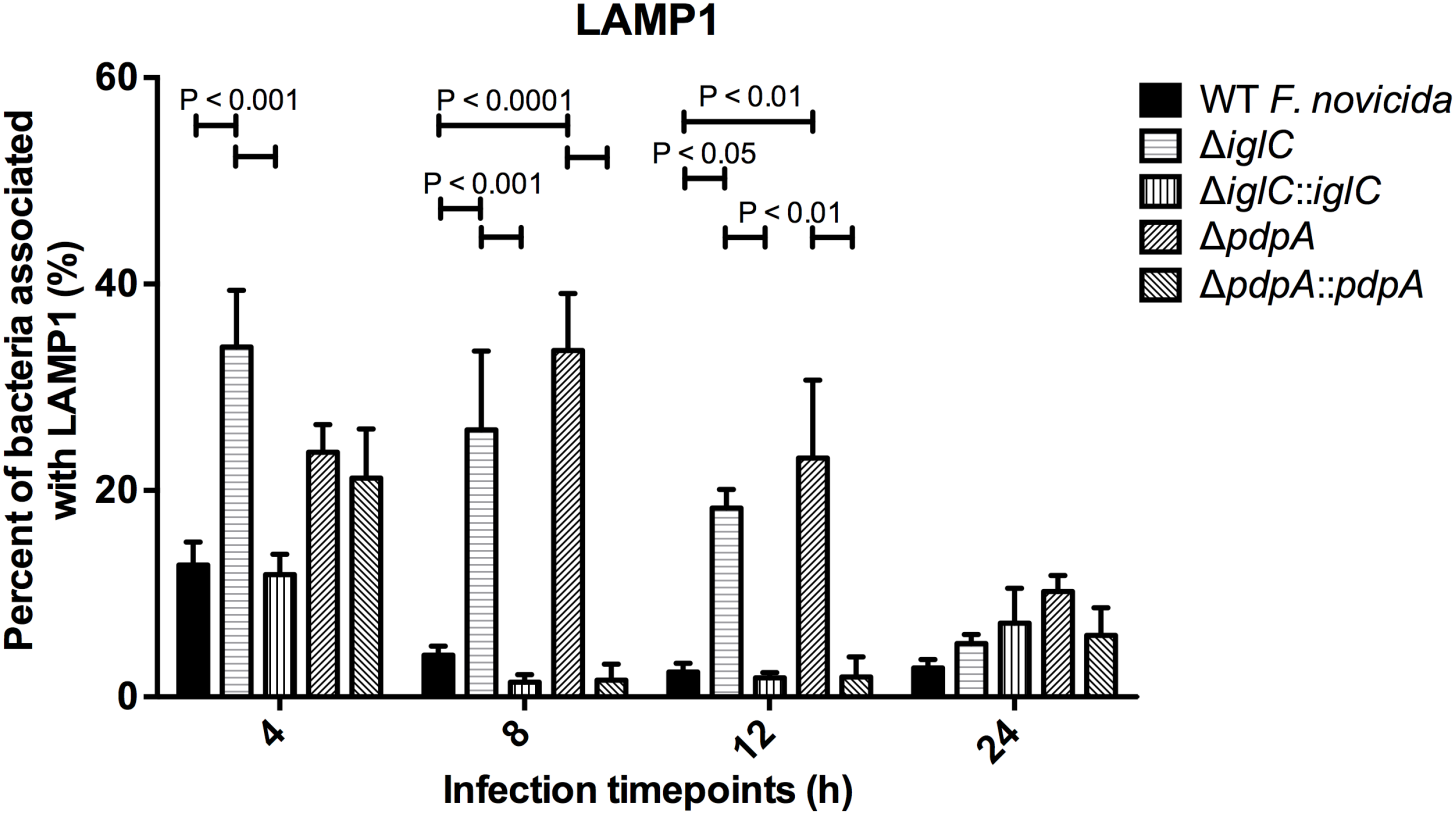
Proportion of *F. novicida* associated with LAMP1 during murine hepatocyte infections. (A) wild-type *F. novicida*, deletion mutants (Δ*pdpA*, Δ*iglC*) and complements strains (Δ*pdpA*::*pdpA*, Δ*iglC*::*iglC*) invaded BNL CL.2 cells for 3 h, after which extracellular bacteria were washed off and then killed with gentamicin (100 µg mL^−1^). Subsequently, samples were exposed to low gentamicin concentration until the experimental endpoint was reached. Image stacks were assembled and used to determine the frequency of LAMP1-associated bacteria. For time-points 4, 8 and 12 h, between 30 and 50 intracellular bacteria were counted. For the 24 h time-point, more than 50 intracellular bacteria were counted. Error bars, S.E.M. (n = 3).

We found that there was a significant increase in the number of bacteria associated with LAMP1 when Δ*iglC* [33.9%, 4 h; 25.9%, 8 h; 18.3%, 12 h] was compared to wild-type *F. novicida* [12.8%, 4 h; 4.0%, 8 h; 2.4%, 12 h] up to 12 h PI (Figure 8). Similarly Δ*pdpA* (33.6%, 8 h; 23.1%, 12 h) showed a significant increase in localization events around *F. novicida* between 8 and 12 h PI when compared to wild-type *F. novicida* (Figure 8). Although there was a was a delay in the phenotypic effects of the *pdpA* complementation until the 8 h time-point, the observation of increased association of LAMP1 around the mutant *F. novicida* suggests that those microbes were impaired in their ability to break out of the FCV, thus remaining in the vacuolar compartment.

## Discussion

Research on the sub-cellular events underlying *Francisella* pathogenesis has primarily concentrated on phagocytic cell infections, while those involved in non-phagocytic epithelial cell infections have remained largely unexplored. This has occurred despite epithelial cells being a primary site of infection and carrying a considerable bacterial load [15], [63], [64], [72]. Given the FPI’s importance for phagocytic cell infections, we began by investigating whether two of the FPI components, IglC and PdpA, affected bacterial entry and replication in epithelial cells. In order to examine this, we used *iglC* and *pdpA* deletion mutants from 2 *Francisella* bacteria; *F. novicida* and *Francisella* LVS. We initially found that both *iglC* and *pdpA* were required for bacterial internalization into liver and lung epithelial cells. To investigate a potential mechanism responsible for the replication impedance that the Δ*iglC* and Δ*pdpA* mutants showed, we turned to the FCVs. *Francisella* is known to rapidly replicate in the cytosol of macrophages and both IglC and PdpA are needed for that process to occur efficiently. By using a common marker for FCVs, LAMP1, we found that LAMP1 localization to Δ*iglC* and Δ*pdpA F. novicida* was maintained long after the wild-type bacteria had shed the LAMP1 protein, suggesting that the FCVs were maintained for a longer duration during the mutant infections as compared to the wild-type bacteria. This is consistent with past epithelial [55] and phagocyte studies [38], [42]. The increased time housed within the FCVs could conceivably impede replication during that time, thus contributing to the decreased bacterial levels seen with the mutants. Although LAMP1 localization was essentially gone by 24 hours in all samples, the delay in FCV breakdown could explain the significant differences seen throughout the 48 h time-points. If FCV escape is inhibited there is the possibility that gentamicin that was added to kill the extracellular *Francisella* could have accumulated within endosomes and fused with the FCVs to kill the FCV contained bacteria. However in other systems where the influence of gentamicin on epithelial cell infections had been assayed this was found to not be the case [73]. In a study that looked directly at this possibility Martinez-Moya and co-workers found that in epithelial cells infected with *Salmonella* Typhimurium for up to 72 hours and had gentamicin in the extracellular milieu, gentamicin did not accumulate in intracellularly [73]. This was contrary to phagocytic dendritic cells, which did have gentamicin accumulation [73].

*Francisella* use different strategies to gain entry into non-phagocytic and phagocytic cells. During phagocytic cell (macrophage) infections, *Francisella* are engulfed by host cells through phagocytic mechanisms [74]–[76]. In contrast to macrophage invasion, we previously found that *F. novicida* and *F. tularensis* LVS utilize clathrin-dependent mechanisms together with cholesterol- to enter hepatocytes [15]. The additional strategy of macropinocytosis has been met with controversy as one group claims an involvement [77], whereas others exclude the possibility [15], [55]. Worth noting is that clear actin-based membrane ruffling at sites of bacterial entry that are required for macropinocytosis have never been documented.

During *Francisella* infections, nearly all of the genes within the FPI are necessary for full virulence *in vivo* as well as for replication within macrophages [26], [28], [32], [34], [50], [78]–[82], but are not required for entry during phagocytic cell infections [27]. We speculate that the FPI does not significantly influence the phagocytic process and thus does not alter other bacterial surface molecules recognized by host phagocytic receptors. Conversely, when *Francisella* invade epithelial cells, our findings suggest that both IglC and PdpA are required for efficient *Francisella* internalization, this further suggests that these components could somehow influence the clathrin-based internalization processes occurring in the host. Whether this alters bacterial ligands, host receptors, or intracellular host internalization mechanisms remains to be elucidated. IglC has been implicated as a component of the *Francisella* T6SS itself [27] and evidence from other systems has indicated that the T6SS can influence bacterial invasion. Studies on *Campylobacter jejuni* and *Pseudomonas aeruginosa* have shown that these microbes utilize T6SSs to enhance bacterial invasion into epithelial cells [83], [84]. Because IglC and PdpA have been detected within the cytoplasm of macrophages, it is conceivable that they could also act as T6SS-dependent effectors [37]. However, the binding partners and precise functions of those factors remain unknown. Because Δ*iglC* and Δ*pdpA* mutants retained some ability to invade, we surmise that *Francisella* employ additional mechanisms for entry into epithelial cells.

An inconsistency that we are faced when we examined bacterial invasion was that while the Δ*pdpA*::*pdpA and* Δ*iglC*::*iglC F. novicida* both had about 3 times the CFUs at 4 h over the mutant strains (Figure 1) and about double the number of cells invaded when examined microscopically, the Δ*iglC*::*iglC* complement did not increase invasion levels significantly over Δ*iglC* when tested by differential staining. What factors could have influenced this? Potential reasons for this could lie in the abundance, proper localization or proper orientation of IglC in the microbe when ectopically expressed. These features could have influenced individual invasion events, while not impeding replication once the bacteria had invaded. Because the 4 h CFU-based invasion assays assessed both the invasion and early stages of replication while the 24 h microscopic assays only examined the number of invaded cells, without addressing the number of individual bacterial cells within each host cell, this could have also compounded our differing complementation results.

Even though others have used centrifugation to force the attachment of *Francisella* to the cell surface thereby allowing bacterial invasion to be assessed at earlier time-points and to “synchronize” the infections [21], [42], [55], [85], we would prefer to not introduce additional variables into the experiments and thus not force bacterial contact with the host cells if this can be avoided. Consequently, we allowed *F. novicida* to naturally contact the cell surface and elicit its own internalization. We are confident that the vast majority of the bacteria we measured at the 4 h time-point are those of invaded bacteria, because significant bacterial replication is not detected until >4 h post-infection, even when centrifugation is used [21], [42], [70]. However, when we used this infection approach on *F. tularensis* LVS, we were unable to detect *F. tularensis* LVS invasion at 4 h. Thus, in order to obtain sufficient invasion, we found that we needed to centrifuge the bacteria onto the cells. To ensure that only intracellular bacteria were counted, we performed concurrent infections in order to detect that no *Francisella* appeared on plates in the absence of epithelial cells (to mimic the extracellular bacteria present during the invasion assays). This indicated to us that the gentamicin was effective at killing any extreacellular *Francisella* (data not shown).

Our work studying the influence of two of the FPI genes, IglC and PdpA, during *Francisella* epithelial cell infections has highlighted important differences in epithelial versus macrophage internalization, while demonstrating that *Francisella* replication is likely governed through similar mechanisms regardless of the cell-type infected. This study provides a framework for elucidating the detailed roles of individual FPI components in both the internalization and replicative phases of the bacterial lifecycle and will ultimately demonstrate how host epithelial cell processes can be subverted by foreign proteins.

## Supporting Information

Figure S1 **Phase and fluorescence micrographs were taken of uninfected and** ***F. novicida*** **infected hepatocytes at 24 h PI.** Mouse BNL CL.2 cells were infected with wild-type *F. novicida* and mutants Δ*iglC*, Δ*iglC::iglC,* Δ*pdpA* and Δ*pdpA::pdpA* for 22 h. Afterwards, samples were washed, treated with gentamicin for 2 h, and then fixed with 3% paraformaldehyde. Fixed samples were prepared using an immunolocalization technique that can differentiate extracellular (green and red co-localization, arrows) and intracellular bacteria (green only, arrowheads). Each fluorescence image represents a superimposed ‘maximum intensity’ Z-projection image around the cell nucleus (blue). Scale bar = 10 µm.
(TIF)

Table S1 **Bacterial doubling time during the early intracellular stages in hepatocytes.**
(DOCX)
