# Supplementary figures and images for: IglC and PdpA Are Important for Promoting Francisella Invasion and Intracellular Growth in Epithelial Cells
Source: PLoS One. 2014 Aug 12;9(8):e104881. doi: 10.1371/journal.pone.0104881 (PMC4130613; doi:10.1371/journal.pone.0104881)

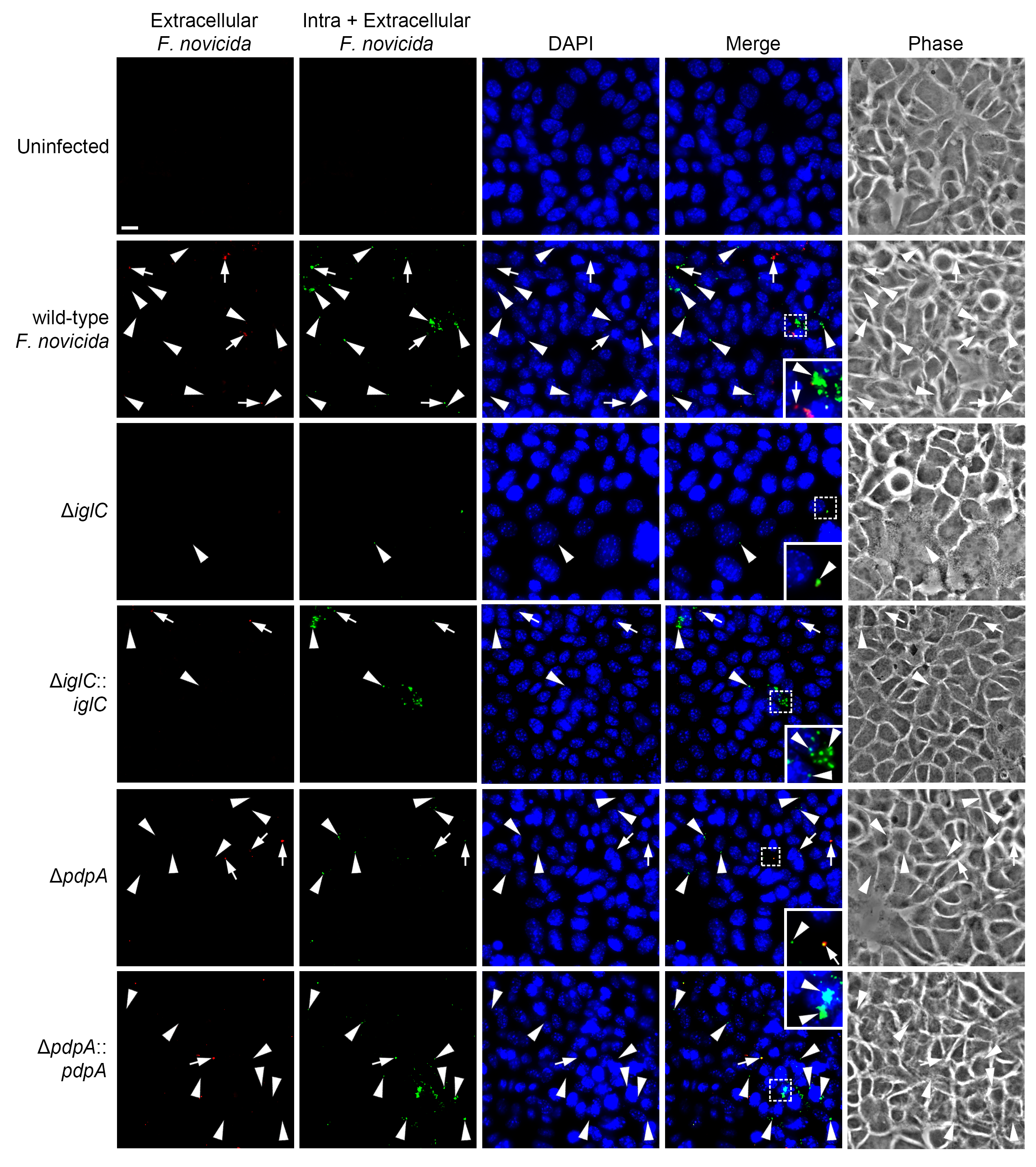

Supplement: Figure S1 — Phase and fluorescence micrographs were taken of uninfected and F. novicida infected hepatocytes at 24 h PI. Mouse BNL CL.2 cells were infected with wild-type F. novicida and mutants ΔiglC, ΔiglC::iglC, ΔpdpA and ΔpdpA::pdpA for 22 h. Afterwards, samples were washed, treated with gentamicin for 2 h, and then fixed with 3% paraformaldehyde. Fixed samples were prepared using an immunolocalization technique that can differentiate extracellular (green and red co-localization, arrows) and intracellular bacteria (green only, arrowheads). Each fluorescence image represents a superimposed ‘maximum intensity’ Z-projection image around the cell nucleus (blue). Scale bar = 10 µm. (TIF) [file pone.0104881.s001.tif]
