# Supplementary material for: IglC and PdpA Are Important for Promoting Francisella Invasion and Intracellular Growth in Epithelial Cells
Source: PLoS One. 2014 Aug 12;9(8):e104881. doi: 10.1371/journal.pone.0104881 (PMC4130613; doi:10.1371/journal.pone.0104881)
Supplement: Table S1 — Bacterial doubling time during the early intracellular stages in hepatocytes. (DOCX) [file pone.0104881.s002.docx]

| Bacteria | Average doubling time (h) | | |
| --- | --- | --- | --- |
|  | **4 to 8h** | **8 to 12 h** | **12 to 24 h** |
| WT *F. novicida* | 1.16 | 1.61 | 4.44 |
| Δ*pdpA* | 8.65 | -5.38 | -14.26 |
| Δ*iglC* | 9.44 | -185.02 | -8.91 |
| Δ*pdpA*::*pdpA* | 1.53 | 1.76 | 5.05 |
| Δ*iglC*::*iglC* | 1.41 | 1.96 | 4.49 |

Table S1. Bacterial doubling time during the early intracellular stages in hepatocytes
